# A novel hierarchical Pt- and FTO-free counter electrode for dye-sensitized solar cell

**DOI:** 10.1186/1556-276X-9-202

**Published:** 2014-05-01

**Authors:** Xing Zhao, Meicheng Li, Dandan Song, Peng Cui, Zhirong Zhang, Yan Zhao, Chao Shen, Zhaohuang Zhang

**Affiliations:** 1 State Key Laboratory of Alternate Electrical Power System with Renewable Energy Sources, School of Renewable Energy, North China Electric Power University, Beijing 102206, China; 2 School of Energy Power and Mechanical Engineering, North China Electric Power University, Beijing 102206, China; 3 Suzhou Institute, North China Electric Power University, Suzhou 215123, China; 4 Chongqing Materials Research Institute, Chongqing 400707, China

**Keywords:** TiO_2_ nanoparticles, PEDOT, PSS, Dye-sensitized solar cells, Counter electrode, Composite film

## Abstract

A novel hierarchical Pt- and FTO-free counter electrode (CE) for the dye-sensitized solar cell (DSSC) was prepared by spin coating the mixture of TiO_2_ nanoparticles and poly(3,4-ethylenedioxy-thiophene):poly(styrenesulfonate) (PEDOT:PSS) solution onto the glass substrate. Compared with traditional Pt/FTO CE, the cost of the new CE is dramatically reduced by the application of bilayer TiO_2_-PEDOT:PSS/PEDOT:PSS film and the glass substrate. The sheet resistance of this composite film is 35 Ω sq^−1^ and is low enough to be used as an electrode. The surface morphologies of TiO_2_-PEDOT:PSS layer and modified PEDOT:PSS layer were characterized by scanning electron microscope, which shows that the former had larger surface areas than the latter. Electrochemical impedance spectra and Tafel polarization curves prove that the catalytic activity of TiO_2_-PEDOT:PSS/PEDOT:PSS/glass CE is higher than that of PEDOT:PSS/FTO CE and is similar to Pt/FTO CE's. This new fabricated device with TiO_2_-PEDOT:PSS/PEDOT:PSS/glass CE achieves a high power conversion efficiency (PCE) of 4.67%, reaching 91.39% of DSSC with Pt/FTO CE (5.11%).

## Background

Dye-sensitized solar cells (DSSCs) have attracted considerable interests due to their simpler fabrication and low production costs compared with conventional silicon-based solar cells [1,2]. A traditional DSSC consists of a transparent photoanode with dye-sensitized mesoporous thin-film-like TiO_2_ or ZnO, I^−^/I_3_^−^ redox electrolyte, and a counter electrode (CE) with a catalytic layer deposited on FTO substrate. As one of the most crucial components of DSSC, the CE works as a catalyst for the reduction of I_3_^−^ to I^−^, and the materials used in catalytic layer and conductive substrates significantly affect the performance and costs of the DSSCs. Platinized FTO is the most common material for CE as it has good conductivity and high catalytic activity. However, noble metal platinum is expensive, scarce, and easy to be eroded by the I^−^/I_3_^−^ electrolyte [3,4]. Moreover, the Pt catalytic layer is usually prepared by thermal annealing or electrodeposition method, and both methods require high temperature (450°C), which is beyond the sustaining ability of plastic substrates to realize the flexible DSSCs. The common FTO substrates are very expensive and hard, also preventing the production of flexible DSSCs. Therefore, it is imperative to develop Pt- and FTO-free CEs with low cost and good catalytic activity for DSSCs.

Many reported materials have been used as the substitute for Pt-based CEs like conductive polymers (polyaniline [5], ploypyrrole [6], poly(3,4-ethylenedioxy-thiophene) (PEDOT) [7], carbon materials (graphene [8], carbon black [9], carbon nanotube [10], etc.), and most of them have lower catalytic activity than Pt [11]. In order to achieve a cost-effective Pt-free CE, PEDOT:PSS has attracted much attention because of good catalytic activity, better film-forming property, low cost, and easy coating [12-14]. Modified PEDOT:PSS has potential to replace TCO in organic electronics for its high conductivity [15]. Though with many of strengths, the catalytic ability of DSSC with PEDOT:PSS/FTO CE still exists a distance from Pt/FTO CE and needs to be further improved.

Consequently, in this work, a hierarchical TiO_2_-PEDOT:PSS/PEDOT:PSS/glass CE was used in the fabrication of DSSC. The TiO_2_-PEDOT:PSS layer was fabricated utilizing the mixture of PEDOT:PSS and TiO_2_ nanoparticles. The neat PEDOT:PSS layer acts as a high conductive electrode in order to develop charge passageway. This hierarchical TiO_2_-PEDOT:PSS/PEDOT:PSS/glass CE performed better catalytic activity than the PEDOT:PSS/FTO CE, and as a result, the DSSC using TiO_2_-PEDOT:PSS/PEDOT:PSS/glass CE also performs good photovoltaic properties.

## Methods

### Preparation of TiO_2_ photoanodes

TiO_2_ paste was blade-coated on FTO substrates and subsequently sintered at 450°C for 30 min. After cooling down to room temperature, the samples were put into 40 mmol/L TiCl_4_ solution at 70°C for 30 min and then sintered at 450°C for 30 min. Finally, after cooling down to 80°C, the as-prepared TiO_2_ photoanodes were soaked in the ethanol solution of N719 dye for 24 h.

### Preparation of the counter electrodes

In total, we have prepared four kinds of CEs, including Pt/FTO, PEDOT:PSS/FTO, TiO_2_-PEDOT:PSS/FTO, and TiO_2_-PEDOT:PSS/PEDOT:PSS/glass. The Pt/FTO CE was prepared by spraying H_2_PtCl_6_ solution on the pre-cleaned FTO substrate and subsequently sintered at 450°C for 15 min. The PEDOT:PSS/FTO and TiO_2_-PEDOT:PSS/FTO CEs were fabricated by spin coating PEDOT:PSS (Clevios PH 1000, purchased from Heraeus, Hanau, Germany) solution and TiO_2_-PEDOT:PSS solution onto FTO substrates, respectively. The TiO_2_-PEDOT:PSS/PEDOT:PSS/glass was obtained by spin coating PEDOT:PSS mixed with 6% volume of ethylene glycol (EG) on glass substrate (5,000 rpm/s for 30 s) and sintered at 120 °C for 15 min. This process was repeated four times. Then, the TiO_2_-PEDOT:PSS (40 mg P25 powder added in 1 ml PEDOT:PSS solution) solution was spin-coated on top of the PEDOT:PSS layer at 1,000 rpm/s for 40 s and sintered at 120°C for 15 min. Finally, the resultant substrates were immediately put into EG for 30 min and then dried in the oven at 120°C for 15 min.

### Fabrication and characterization of DSSCs

The processed TiO_2_ photoanodes have an active area of 0.16 cm^2^, and these prepared CEs were assembled together with 60-μm surlyn film, respectively. The I^−^/I_3_^−^ electrolyte was injected through the interspace and sealed with paraffin.

The sheet resistance of the catalytic layers was measured using a four-probe tester (model RTS-8, Four Probe TECH, Guangzhou, China). The surface morphologies of CEs were scanned by field emission scanning electron microscope (quanta 200 F, FEI, OR, USA). Electrochemical impedance spectroscopy (EIS) and Tafel polarization curves were measured using an electrochemical workstation (model CHI600, CH Instruments, Inc., Austin, TX, USA) at room temperature. The current density-voltage characteristics of photocurrent density-photovoltage were simulated at AM 1.5G illumination (100 mV cm^−2^, XES-301S, SAN EI, Osaka, Japan) and recorded by a Keithley source meter (Keithley, Cleveland, OH, USA).

## Results and discussion

The sheet resistance of different CEs, PEDOT:PSS/FTO CE, TiO_2_-PEDOT:PSS/FTO CE, TiO_2_-PEDOT:PSS/PEDOT:PSS/glass CE, and Pt/FTO CE, is 6.3, 7.5, 35, and 7.2 Ω sq^−1^, respectively. Though the sheet resistance of TiO_2_-PEDOT:PSS/PEDOT:PSS/glass CE is larger than that of TiO_2_-PEDOT:PSS/FTO CE and Pt/FTO CE, it is still qualified, i.e., the sheet resistance below 100 Ω sq^−1^ can be used as electrode [16,17].

**Figure 1:**
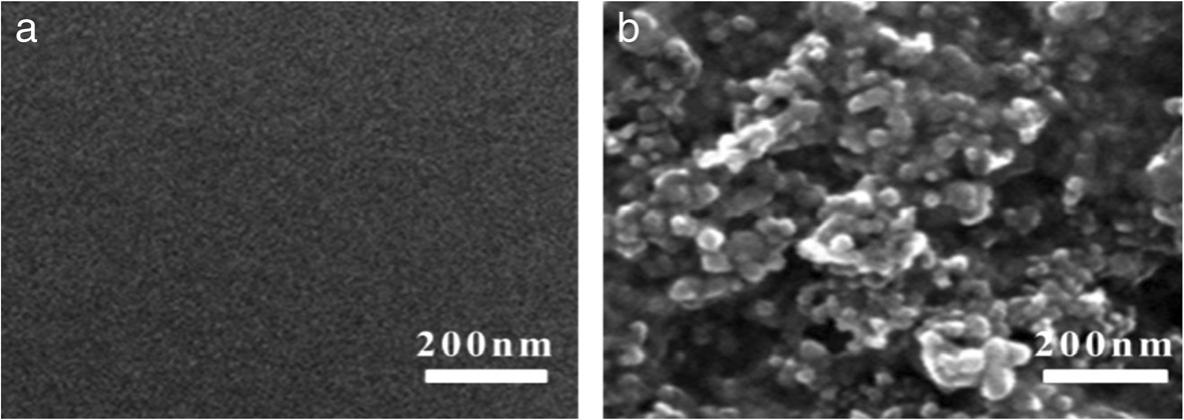
**SEM images of PEDOT:PSS film (a) and TiO**_**2**_**-PEDOT:PSS composite film (b).**

The surface morphologies of pristine PEDOT:PSS film and TiO_2_-PEDOT:PSS composite film are depicted in Figure 1a,b, respectively. As is shown in the two images, the surface of modified PEDOT:PSS film is almost smooth, while the TiO_2_-PEDOT:PSS composite film is rough and has a large surface area which is good for catalytic reduction of I_3_^−^. In TiO_2_-PEDOT:PSS composite film, as shown in Figure 1b, the thin catalytic layer is composed of TiO_2_ nanoparticles, and their diameter ranges from 20 to 50 nm. These nanoparticles are uniformly dispersed in PEDOT:PSS, forming a network structure, beneficial for electron conduction. Therefore, the performance of DSSCs with TiO_2_-PEDOT:PSS/PEDOT:PSS/glass CEs could be greatly improved by the addition of TiO_2_ nanoparticles.

**Figure 2:**
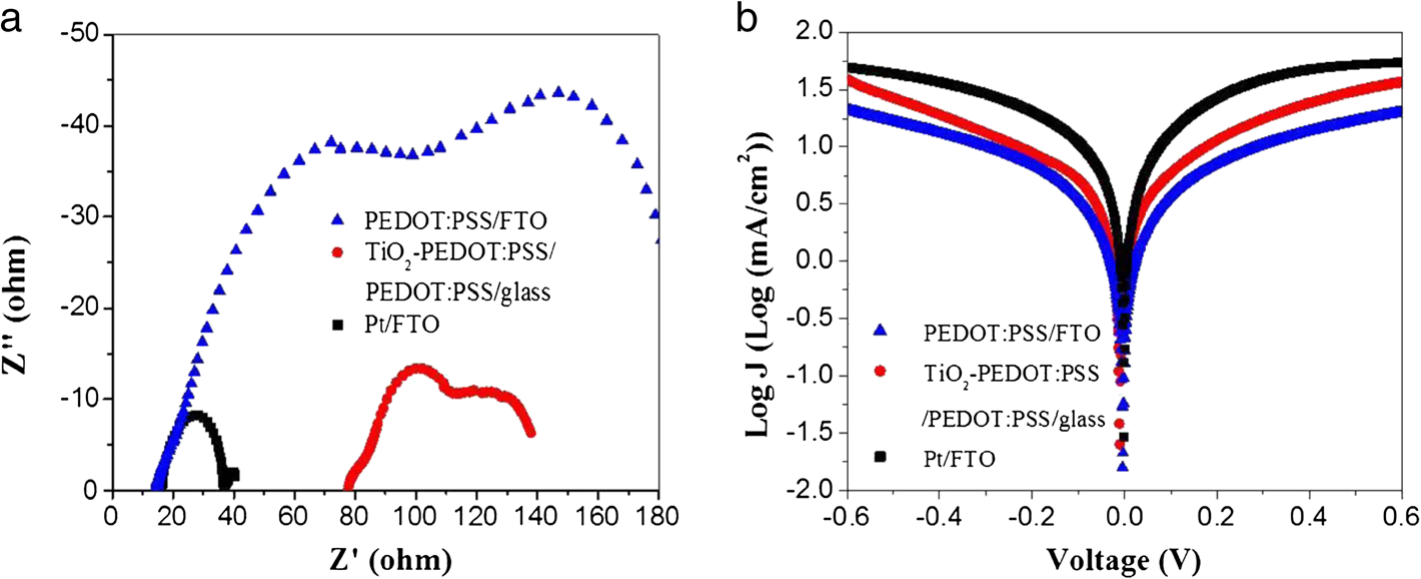
**Electrochemical characters.** Nyquist plots **(a)** and Tafel polarization curves **(b)** of DSSCs based on PEDOT/FTO CE, TiO_2_-PEDOT:PSS/PEDOT:PSS/glass CE, and Pt/FTO CE.

A typical EIS spectrum for a DSSC exhibits three semicircles in the Nyquist plot, as is shown in Figure 2a. Traditionally, the first semicircle in high-frequency region corresponds to charge transfer resistance (*R*_ct_) of the CE/electrolyte interface, while the second semicircle in the middle-frequency region represents charge transfer and recombination resistance in the TiO_2_/dye network [18,19]. The low-frequency semicircle is attributed to the Nernst diffusion impedance of the I^−^/I_3_^−^ redox couple. From Figure 2a, we can obviously see that the spectra of TiO_2_-PEDO:PSS/PEDO:PSS/glass CE has a smaller semicircle than that of the POEDT:PSS/FTO CE, which indicates that TiO_2_-PEDO:PSS/PEDO:PSS/glass CE has a better catalytic activity than POEDT:PSS/FTO CE. The simulated values of series resistance (*R*_s_), charge tansfer resistance (*R*_ct_), and diffusion element (*Z*_w1_) of corresponding cells calculated by Zview software are shown in Table 1. The simulated *R*_ct_ and *Z*_w1_ of TiO_2_-PEDO:PSS/PEDO:PSS/glass CE (1.51 and 4.02 Ω cm^2^, respectively) are lower than those of PEDOT:PSS/FTO CE (4.47 and 11.28 Ω cm^2^, respectively), indicating that the addition of TiO_2_ nanoparticles greatly improves the catalytic activity for the redox reaction. The *R*_s_ value of TiO_2_-PEDOT:PSS/PEDOT:PSS/glass CE is higher than that of PEODT:PSS/FTO CE due to a lower conductivity of PEDOT:PSS layer than that of FTO substrate, and the result is in accordance with the conclusion from the sheet resistance. However, the *R*_ct_ of TiO_2_-PEDOT:PSS/PEDOT:PSS/glass composite CE is lower than that of Pt/FTO CE (5.73 Ω cm^2^) which is opposite to the traditional standpoint that a smaller *R*_ct_ may lead to a higher fill factor (FF) and *η* in photovoltaic performance. However, for TiO_2_-PEDOT:PSS/PEDOT:PSS/glass CE, the charge transfer of the CE/electrolyte interface is mainly illustrated by the second semicircle of the spectra. Similar findings have been reported by He et al. [20] and Roy-Mayhew et al. [8], and they contend that the second semicircle is due to the electrolyte/CE interface.

**Table 1:** **Electrochemical impedance spectra (EIS) parameters of PEDOT/FTO CE, TiO**_**2**_**-PEDOT:PSS/PEDOT:PSS/glass CE, and Pt/FTO CE**

**Counter electrode**	***R***_**s **_**(Ω cm**^**2**^**)**	***R***_**ct **_**(Ω cm**^**2**^**)**	***Z***_**W1 **_**(Ω cm**^**2**^**)**
PEDOT:PSS/FTO	4.22	4.47	11.28
TiO_2_-PEDOT:PSS/PEDOT:PSS/glass	23.26	1.51	4.02
Pt/FTO	4.91	5.73	-

Furthermore, Tafel polarization curves were carried out on the same dummy cells used in EIS measurement to investigate the interfacial charge transfer properties of CE/electrolyte, and the corresponding results are shown in Figure 2b. The exchange current (*J*_0_) = 0.58 mA, calculated from the intersection of the linear cathodic and anodic Tafel polarization curves [16,21], was derived from the TiO_2_-PEDOT:PSS/PEDOT:PSS/glass composite film and higher than that of PEDOT:PSS/FTO film (0.14 mA). Correspondingly, the catalytic activity of TiO_2_-PEDOT:PSS/PEDOT:PSS/glass composite CE is much higher than that of PEDOT:PSS/glass CE, which demonstrates that the big surface area of TiO_2_ nanoparticles enhances the reduction of I_3_^−^ to I^−^ remarkably. Though the *J*_0_ of TiO_2_-PEDOT:PSS/PEDOT:PSS/glass composite CE is smaller than that of Pt/FTO CE (1.2 mA), the former still exhibits superior catalytic activity and has great potential to act as CE for DSSC.

**Figure 3:**
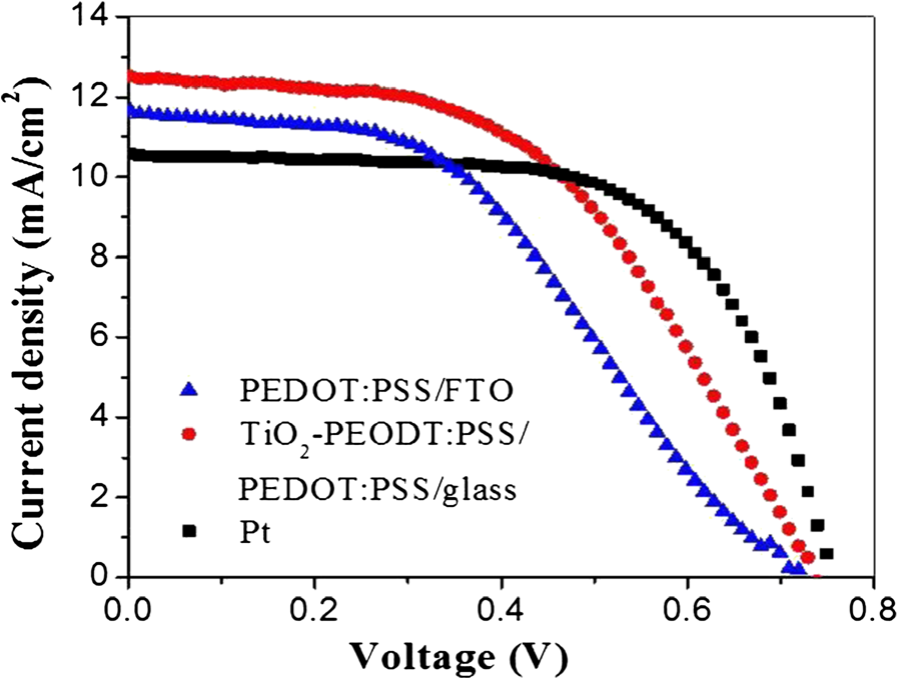
**Current density-voltage (*J *-*V *) characteristics of DSSCs based on PEDOT/FTO, TiO**_**2**_**-PEDOT:PSS/PEDOT:PSS/glass, and Pt/FTO CEs.**

Figure 3 presents the photocurrent density-voltage (*J*-*V*) curves of DSSCs using PEDOT:PSS/FTO CE, TiO_2_-PEDOT:PSS/PEDOT:PSS/glass CE, and Pt/FTO CE, respectively, and the related photovolatic parameters are shown in Table 2. There is little difference in *V*_oc_ values of these three cells. The FF of the DSSC with PEDOT:PSS/FTO CE is just 0.43 because of the poor catalytic activity of PEDOT:PSS solution. After modified by the TiO_2_ nanoparticles, the DSSC with TiO_2_-PEDOT:PSS/PEDOT:PSS/glass CE has obtained higher FF of 0.51 and thus higher *η* = 4.67% (increasing 22% compared with 3.64% for the DSSC with PEDOT:PSS/FTO CE). This is mainly due to the reduced charge transfer resistance and porous diffusion impedance because of the large electrochemical surface area in the porous TiO_2_-PEDOT:PSS layer. Compared with DSSC based on Pt/FTO CE, the one with TiO_2_-PEDOT:PSS/PEDOT:PSS/glass CE has lower FF, but its overall efficiency has already reached 91.39% of the one with Pt/FTO CE. It is noticeable that the performance of TiO_2_-PEDOT:PSS/PEDOT:PSS layers can befurther enhanced by optimazation of their weight ratio and the film thicknesses, referring to the previous studies using TiO_2_-PEDOT:PSS/FTO CE [22]. With such an excellent performance, the TiO_2_-PEDOT:PSS/PEDOT:PSS/glass CE has great potential to be a substitute for Pt- and FTO-based CEs which are very expensive and account for a large part of the cost. At the same time, due to the low preparation temperature, the TiO_2_-PEDOT:PSS/PEDOT:PSS composite film can be applied in flexible cells and make them more functional and lightweight.

**Table 2:** The performances of dye-sensitized solar cells with different CEs measured under an AM 1.5G illumination

**Counter electrode**	***V***_**oc **_**(V)**	***J***_**sc **_**(mA cm**^**−2**^**)**	**FF**	***η *** **(%)**
PEDOT:PSS/FTO	0.72	11.63	0.43	3.64
TiO_2_-PEDOT:PSS/PEDOT:PSS/glass	0.73	12.45	0.51	4.67
Pt/FTO	0.75	10.54	0.63	5.11

## Conclusions

In summary, we utilize a facile wet method to fabricate a novel hierarchical Pt- and FTO-free CE for the dye-sensitized solar cell. It is found that the TiO_2_ doped PEDOT:PSS catalytic activity layer will dramatically affect the electrochemical properties of the final device. By adjusting the composition of TiO_2_, the properties of CE have been optimized preliminarily. Because of the large active area of TiO_2_ nanoparticles, the proposed composite CE shows excellent enhancement in the conductivity and the superior catalytic activity for the reduction of I_3_^−^ to I^−^. The conversion efficiency is increased by 22% than that of the DSSC with PEDOT:PSS/FTO CE and is comparable to that of the DSSC with traditional Pt/FTO CE. After further optimization, the TiO_2_-PEDOT:PSS/PEDOT:PSS/glass CE can be more cost-effective, high efficient, and flexible to replace Pt and FTO CEs and more broadly used for future commercial applications.

## Competing interests

The authors declare that they have no competing interests.

## Authors’ contributions

XZ did most of the experiments and drafted the manuscript. ML designed and figured out the research idea and rewrote the paper. DS did part of the research experiments. PC participated in the design of the study. ZrZ, YZ, CS, and ZhZ took part in the discussion of the research. All authors read and approved the final manuscript.

## References

[B1] O'Regan B Grätzel M A low-cost, high-efficiency solar cell based on dye-sensitized colloidal TiO_2_ films Nature 1991 9 737 740

[B2] Grätzel M Photoelectrochemical cells Nature 2001 9 338 344 11713540 10.1038/35104607

[B3] Xu HG Zhang XY Zhang CJ Liu ZH Zhou XH Pang SP Chen X Dong SM Zhang ZY Zhang LX Han PX Wang XG Cui GL Nanostructured titanium nitride/PEDOT:PSS composite films as counter electrodes of dye-sensitized solar cells ACS Appl Mater Interfaces 2012 9 1087 1092 22264094 10.1021/am201720p

[B4] Song DD Li MC Bai F Li YF Jiang YJ Jiang B Silicon nanoparticles/PEDOT-PSS nanocomposite as an efficient counter electrode for dye-sensitized solar cells Funct Mater Lett 2013 9 4 1350048

[B5] Li QH Wu JH Tang QW Lan Z Li PJ Lim JM Fan LQ Application of microporous polyaniline counter electrode for dye-sensitized solar cells Electrochem Commun 2008 9 1299 1302

[B6] Bu CH Tai QD Liu YM Guo SS Zhao XZ A transparent and stable polypyrrole counter electrode for dye-sensitized solar cell J Power Sources 2013 9 78 83

[B7] Lee KS Lee HK Wang DH Park NG Lee JY Park OO Park JH Dye-sensitized solar cells with Pt- and TCO-free counter electrodes Chem Commun 2010 9 4505 4507 10.1039/c0cc00432d 20464028

[B8] Roy-Mayhew JD Bozym DJ Punckt C Aksay IA Functionalized graphene as a catalytic counter electrode in dye-sensitized solar cells ACS Nano 2010 9 6203 6211 20939517 10.1021/nn1016428

[B9] Lim J Ryu SY Kim J Jun Y A study of TiO_2_/carbon black composition as counter electrode materials for dye-sensitized solar cells Nanoscale Res Lett 2013 9 227 23672498 10.1186/1556-276X-8-227 PMC3665677

[B10] Huang SQ Sun HC Huang XM Zhang QX Li DM Luo YH Meng QB Carbon nanotube counter electrode for high-efficient fibrous dye-sensitized solar cells Nanoscale Res Lett 2012 9 222 22507398 10.1186/1556-276X-7-222 PMC3441495

[B11] Murakami TN Grätzel M Counter electrodes for DSC: application of functional materials as catalysts Inorg Chim Acta 2008 9 572 580

[B12] Zhang TL Chen HY Su CY Kuang DB A novel TCO- and Pt-free counter electrode for high efficiency dye-sensitized solar cells J Mater Chem A 2013 9 1724 1730

[B13] Chiang CH Wu CG High-efficient dye-sensitized solar cell based on highly conducting and thermally stable PEDOT:PSS/glass counter electrode Org Electron 2013 9 1769 1776

[B14] Chou CS Chou CS Kuo YT Wang CP Preparation of a working electrode with a conducting PEDOT:PSS film and its applications in a dye-sensitized solar cell Adv Powder Technol 2013 9 336 343

[B15] Kim YH Sachse C Machala ML May C Müller-Meskamp L Leo K Highly conductive PEDOT:PSS electrode with optimized solvent and thermal post-treatment for ITO-free organic solar cells Adv Funct Mater 2011 9 1076 1081

[B16] Yue GT Wu JH Xiao YM Lin JM Huang ML Lan Z Fan LQ Functionalized graphene/poly(3,4-ethylenedioxythiophene):polystyrenesulfonate as counter electrode catalyst for dye-sensitized solar cells Energy 2013 9 315 321

[B17] Song DD Li MC Jiang YJ Chen Z Bai F Li YF Jiang B Facile fabrication of MoS_2_/PEDOT-PSS composites as low-cost and efficient counter electrodes for dye-sensitized solar cells J Photoch Photobio A 2014 9 47 51

[B18] Wang Q Moser JE Grätzel M Electrochemical impedance spectroscopic analysis of dye-sensitized solar cells J Phys Chem 2005 9 14945 14953 10.1021/jp052768h 16852893

[B19] Hauch A Georg A Diffusion in the electrolyte and charge-transfer reaction at the platinum electrode in dye-sensitized solar cells Electrochim Acta 2001 9 3457 3466

[B20] He JJ Duffy NW Pringle JM Cheng YB Conducting polymer and titanium carbide-based nanocomposites as efficient counter electrodes for dye-sensitized solar cells Electrochim Acta 2013 9 275 281

[B21] Yan XD Zhang LZ Polyethylene glycol-modified poly(3,4-ethylenedioxythiophene):poly (styrenesulfonate) counter electrodes for dye-sensitized solar cell J Appl Eelctrochem 2013 9 605 610

[B22] Maiaugree W Pimanpang S Towannang M Saekow S Jarernboon W Amornkitbamrung V Optimization of TiO_2_ nanoparticle mixed PEDOT–PSS counter electrodes for high efficiency dye sensitized solar cell J Non-Cryst Solids 2012 9 2489 2495

